# Resistance and Adaptation of Bacteria to Non-Antibiotic Antibacterial Agents: Physical Stressors, Nanoparticles, and Bacteriophages

**DOI:** 10.3390/antibiotics10040435

**Published:** 2021-04-13

**Authors:** Sada Raza, Kinga Matuła, Sylwia Karoń, Jan Paczesny

**Affiliations:** Institute of Physical Chemistry, Polish Academy of Sciences, Kasprzaka 44/52, 01-224 Warsaw, Poland; sraza@ichf.edu.pl (S.R.); Kinga.Matula@ru.nl (K.M.); sylwia.antoninak@gmail.com (S.K.)

**Keywords:** antibacterial agents, resistance, bacterial adaptation, temperature, pressure, electric field, nanoparticles, bacteriophages, mechanism

## Abstract

Antimicrobial resistance is a significant threat to human health worldwide, forcing scientists to explore non-traditional antibacterial agents to support rapid interventions and combat the emergence and spread of drug resistant bacteria. Many new antibiotic-free approaches are being developed while the old ones are being revised, resulting in creating unique solutions that arise at the interface of physics, nanotechnology, and microbiology. Specifically, physical factors (e.g., pressure, temperature, UV light) are increasingly used for industrial sterilization. Nanoparticles (unmodified or in combination with toxic compounds) are also applied to circumvent in vivo drug resistance mechanisms in bacteria. Recently, bacteriophage-based treatments are also gaining momentum due to their high bactericidal activity and specificity. Although the number of novel approaches for tackling the antimicrobial resistance crisis is snowballing, it is still unclear if any proposed solutions would provide a long-term remedy. This review aims to provide a detailed overview of how bacteria acquire resistance against these non-antibiotic factors. We also discuss innate bacterial defense systems and how bacteriophages have evolved to tackle them.

## 1. Introduction

The last decade has shown that antibiotic resistance is one of the most pressing issues in the healthcare system, causing a financial burden on hospitals and societies due to the prolongation of illness and subsequent treatment [1]. Infected individuals also face significant health and economic consequences. This burden is estimated at USD 1.3 to 2.7 billion in the USA and USD 1.5 billion in the EU per year [2].

New strains of superbugs are discovered at an alarming rate due to human negligence of antibiotics (misuse or overuse) and acquisition of new mutations leading to rapidly evolving bacterial strains [3,4]. Spontaneous changes in bacterial genetic material (mutational resistance) confer resistance towards a wide range of antibiotics [5]. It is also proven that bacterial strains take only a few hours to prevent the antibiotic from reaching its destination by modifying drug molecule uptake, sequestering the molecule, and bypassing target sites [5]. Overuse of antibiotics leads to horizontal gene transfer of resistance between different species [5,6]. Potent, unregulated drugs are made available over the counter, further increasing the likelihood of generating more resistant bacterial species [7]. In 2015, the emergence of resistance against colistin was reported [8].

Interestingly, the data on the global spread of gene mcr-1, responsible for the resistance, was published in 2018 [9]. Later, the gene was found in several bacteria such as *Escherichia*, *Salmonella*, *Klebsiella*, *Kluyvera*, *Citrobacter*, and *Cronobacter* [9]. Colistin used to be the drug of last resort—used when all other antibiotics fail to treat an infection. With spreading colistin resistance, we have entered the post-antibiotic era.

To combat multi-drug resistant (MDR) superbugs, a plethora of novel methods are under investigation (e.g., nanotechnology-based), while old and momentarily forgotten strategies (bacteriophages, physical factors) are being revised.

Physical factors, such as UV light, high pressure, and temperature, are used for disinfection, especially in industries with a high risk of microbial contamination [10]. Physical factors are not targeted (they affect all organisms) that vastly limit their application.

Scientists have started exploring the prospects of using nanoparticles (NPs)—either to deliver antimicrobial agents or as antimicrobial substances themselves [11,12]. There are high expectations of the efficient killing of bacteria using nanostructures and their non-chemical mechanisms such as contact killing [13], mechanical puncturing [14], and changes in the local microenvironment via nano ions [15]. In addition, nanoparticles combined with antibacterial agents are also being studied [16].

Since the early 1900s, bacteriophages are employed for medical purposes [17]. Several companies and research laboratories pursue a treatment strategy involving phages in infections caused by *Staphylococcus aureus*, *Pseudomonas aeruginosa*, and *Escherichia coli* [11]. In some countries (e.g., Russia, Georgia, Poland, USA), bacteria that do not respond to conventional antibiotics are treated by phages [18]. In Russia and Georgia, phage-based products are available without a prescription [19]. For instance, *Microgen*, a Russian company, sells phages as liquid preparations or as pills even via the internet [20]. Shall we consider this action as the first step towards a similar overuse as in the case of antibiotics?

**Figure 1:**
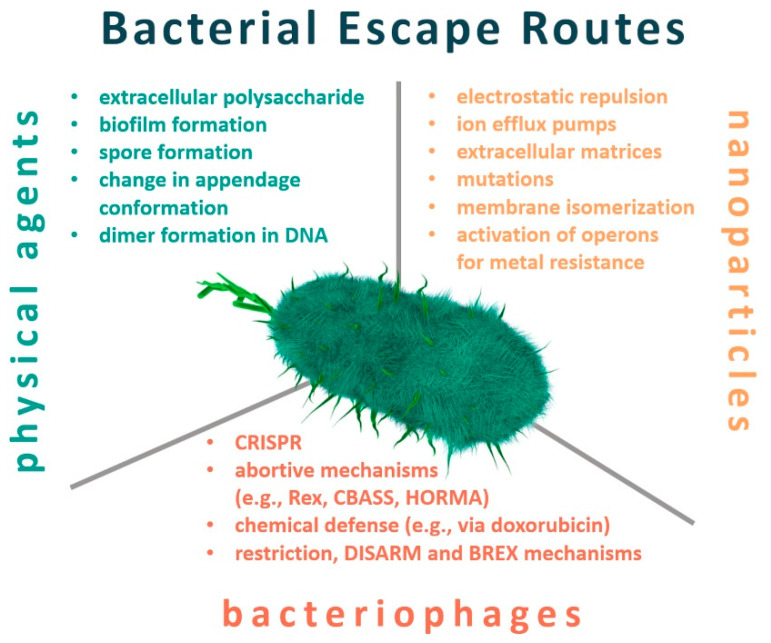
Escape routes utilized by bacteria to deal with non-antibiotic antibacterial agents, i.e., physical factors, nanoparticles, and bacteriophages.

The question remains whether the use of non-antibiotic antibacterial agents is the appropriate alternative to adopt in the post-antibiotic era? If yes, are we prepared to eliminate antibiotics from our healthcare system without causing an outburst of adverse reactions? This review summarizes the reported cases of the appearance of resistance and adaptation (Figure 1) to such non-antibiotic antibacterial agents, with a particular focus on the arms-race between bacteria and bacteriophages.

## 2. Resistance to Physical Factors

Bacteria exist in extreme conditions where temperatures can reach up to 350 °C, in places where hydrostatic pressure comes up to 600–1000 atmospheres or where multiple adverse factors are present. Nevertheless, there are physical factors, especially those not common in nature, that bacteria are vulnerable to. Variations in oxygen concentration and temperature have shown changes in bacterial communities, while the use of pulsed electromagnetic field (PEMF) on NPs decorated with nisin protein presents results similar to electroporation or magnetic hyperthermia [21,22]. Other methods include the use of UV light, high hydrostatic pressure, manosonication, a change in pH, and heat shock [23]. The pH has proven to be a major contributing factor in enhancing the efficiency of antibacterial agents. Several physical factors can enhance antibacterial effects by overcoming multi-drug resistance in bacteria.

Heat, acidic, and alkaline shocks are generally more effective on Gram-positive bacteria due to their cell walls’ constituents. A thinner wall of Gram-negative species makes them more amenable to the effect of disinfectants. Amongst the Gram-negative species, *P. aeruginosa* is the most resistant, owing to the higher degree of crosslinking of the peptidoglycan. The higher degree of murein crosslinking makes the cell wall resistant to rupture [24]. The efficiency of new methods, such as cold atmospheric-pressure plasma (CAP), also relies on bacterial cell wall thickness. Biofilms formed by Gram-positive bacteria that possess a thicker cell wall show greater resistance to CAP [25].

### 2.1. Temperature

Most mesophilic bacteria are eliminated at 55 °C [22]. Thermal treatment irreversibly affects outer and inner membranes, the peptidoglycan cell wall, the nucleoid, nucleic acids, ribosomes, and various enzymes. The higher temperature also damages the cell’s chemiosmotic and transport functions [26].

*Aeromonas* and *Campylobacter* are two of the most temperature-sensitive genera, while *Enterococcus* is thoroughly temperature resistant. The structural difference between Gram-positive and Gram-negative bacteria also makes the prior generally more resistant [26]. High temperatures alter the bacterial cell wall. Gram-negative bacteria experience destabilization of the outer membrane, thereby increasing the permeability barrier that leads to the release of periplasmic proteins [27].

Magnesium ions play an essential role in the stability of ribosomal subunits. Some chaperones also bind directly to the ribosomes to capture the nascent protein chains to assist their folding and prevent aggregation [28]. Higher temperatures damage the cell wall, causing a depletion of magnesium, leading to disruption of ribosomes’ stability [29]. It was established that RNA and ribosomes are more thermo-sensitive than DNA, and this property can thus be further exploited during thermal inactivation [26].

There are cases of resistant bacteria, especially *E. coli*, secreting extracellular polysaccharides such as colonic acid forming a mucoid matrix on the cell surface [23]. Other modes of acquired resistance include adjusting membrane-lipid composition and membrane fluidity via homoviscous adaptation [27]. Processes, such as slow heating, induce heat adaptation via an increase of membrane fluidity. This thermotolerance is achieved by the *de novo* protein synthesis, which is abolished after cooling [30].

### 2.2. UV Light

Another physical factor used to eradicate bacteria is UV light [31]. The properties of the used medium determine the lethality of the UV treatment [28]. Experiments with UV light revealed 4log-inactivation of both, Gram-positive and Gram-negative strains after exposure for 360 min [32].

Devices such as Xenex Germ-Zapping Robot^TM^, are used to produce a high-intensity flashing light (200–320 nm) that passes through the cell wall of bacteria, viruses, and bacterial spores, hence causing damage to DNA, RNA, and proteins by photohydration, photo splitting, photocrosslinking, and photodimerization [33].

In the case of food contaminated with highly resistant bacteria, UV can be used as an alternative to thermal preservation with the advantage of sterilization in mild temperatures [28]. Importantly, it was observed that coliform bacteria, isolated on media containing chloramphenicol, show a lesser ability to transfer resistance after UV irradiation [34].

Although UV light exhibits antibacterial activity, bacteria with pigments such as melanin, scytonemin, and mycosporines form the first defense line and become resistant [35]. Five genes that provide resistance to UV radiation in *E. coli* were identified [35]. Such tolerance to UV light depends upon dormancy of DNA replication at UV exposure: When cells are in a dormant phase, they can evade death by safeguarding their essential components [36]. Physical protection and antioxidant osmoprotectants constitute the second response mechanism for mutagenicity of DNA damage repair in microorganisms. For instance, *Rhodobacter* sp. uses alternate means of energy production to meet energy demands to synthesize molecules that prevent oxidative stress caused by UV [37].

UV radiation directly impacts bacterial DNA due to the reactive oxygen species (ROS) formation and mutagenesis leading to oxidative stress. This is often combated by molecules such as osmoprotectants, inositol or carotenoid pigments [35]. Another method of acquiring tolerance against UV involves selecting mutations arising in different genes pertaining to DNA repair and replication [38].

In Antarctica, certain UV-tolerant bacteria such as *Pseudomonas*, *Hymenobacter*, and *Sphingomonas* have shown the presence of novel photolyases [39]. Intriguingly, Antarctic bacteria also exhibit an antioxidant defense mechanism to prevent oxidative damage linked to the presence of antioxidant enzymes such as catalases that cause detoxification of H_2_O_2_.

UV irradiation directly impacts DNA by the formation of cyclobutene pyrimidine dimers and pyrimidine pyrimidone (6–4) photoproducts [40]. *Deinococcus swuensi* is an example of a highly resistant strain, which shows a unique landscape of differentially expressed genes that distinctly determine resistance to UV radiation compared to commonly observed mechanisms [41].

### 2.3. Pressure

High pressure evokes inactivation through membrane modifications, deactivation of critical enzymes, and protein biosynthesis inhibition [42]. Other effects include protein denaturation by ionization and ionic bonds forming between charged groups on proteins, altering their solubility [43].

However, some bacterial strains are resistant to high pressures and can only be deactivated by the combined action of temperature and pressure [44]. The inactivation of bacterial spores is also carried out by associating heat and/or chemical treatment with pressurization [44]. High-pressure sterilization is a method to kill endospores that are typically resistant to most antibacterial agents. The boiling point is increased by artificially increasing the pressure, thereby killing the endospores [45]. The inactivation mechanism during high-pressure thermal sterilization (HPTS) passes through three states, namely dormancy, activation, and inactivation. With increasing intensity, the extent of inactivation also increases [46].

Depending on the composition and structure of the lipid bilayer of the cell membrane, respiratory proteins, and the membrane-localized signaling system, some bacteria are more resistant to high pressures than others [42]. For instance, spore-forming bacilli strains, such as *Bacillus alvei*, *Bacillus coagulans*, and *Bacillus subtillis*, isolated from pressurized milk, are found to be resistant [47]. This resistance is generally acquired due to treatment with high-pressure pulses or short-duration treatments, often suppressing metabolic pathways or adaptation of new ones when exposed to high pressure [48]. The pH also plays a vital role in acquiring resistance among several species. A case in point is *Bacillus subtilis* that shows a higher resistance at neutral or slightly basic pH [43].

Another example of resistant bacteria includes *Lactobacillus sanfranciscensis* that only shows tolerance to high pressure when pre-incubated at a pressure range of 0.1–120 MPa [48]. While experimenting with the coastal bacterial community, certain species such as *Alphaproteobacteria*, *Gammaproteobacteria*, *Actinobacteria*, and *Flavobacteria* showed elevated resistance to high pressure than others, such as *Epsilonproteobacteria*, and these species are phylogenetically similar to isolates from deep-sea environments [44].

### 2.4. Electric Field

In the literature, two main types of experiments on the influence of the electromagnetic field on bacteria can be distinguished. Bacteria are subjected to the electromagnetic field of constant frequency and variable intensity of electric strength over time. Typically, a flask with bacterial suspension is placed inside the solenoid, and the number of bacteria is measured over time. Such investigations are driven by the dominant magnetic component of the electromagnetic field during bacteria exposure. The second type of study comprises experiments where bacteria are subjected to the pulsed electric field. The bacteria are placed between two metal plates, and then voltage pulses are applied between two electrodes. In consequence, cells are subjected to the pulsed electric field, and the magnetic component is negligible.

Electroporation is a well-known method utilized in microbiology. Upon application of the pulsed electric field (PEF), holes are created in the cell wall, and thus, its permeability increases [49]. It is used, among others, in biotechnology to introduce a foreign DNA into the interior of bacteria [50]. In such a case, one usually tunes PEF parameters so that creating holes is reversible [49]. The amplitude, pulse number, and pulse duration form are the factors that determine inactivation, especially for species such as *E. coli* and *Lactobacillus acidophilus* [51]. The mechanism of electroporation is still not well understood [52], but the parameters at which this phenomenon occurs are known. The most important is the minimum value of the strength of the electric field strength, which is used to expose the bacterium and induce reversible electroporation. For *E. coli BL21* this value is 3.65 ± 0.09 kV/cm [53]. The most exciting fact is that antibiotic susceptibility is enhanced by the application of PEF, as concluded by the increased zones of growth inhibition [51]. Currently, PEF is also evaluated as a new method for cancer treatment [54].

The most common effects of exposure to PEF include swelling of bacterial cells, increased roughness on the outer membrane, noticeable stiffness, and a loss of hydrophobicity [55]. Other distortions include morphological and mechanical alterations of the cell wall and partial destruction of coat protein structures [55]. In some bacteria, e.g., *Bacillus pumilus*, cell wall, and coat structure are directly involved in the process of inactivation via PEF [55]. In some bacterial species, such as *Streptococcus thermophilus*, exposure to the electric field increases the cell membrane’s permeabilization, which ultimately leads to the reduction of the lag phase [56]. In the case of *Pseudomonas fluorescens*, the electric potential also impacts the conformation of surface appendages, causing steric repulsion allowing the cells to overcome the electrostatic energy barrier [57]. As opposed to most vegetative bacteria, it is noteworthy that bacterial spores are highly resistant to the effect of electric pulse, such as in the case of *B. subtilis* [58].

Pulsed electric field is being popularly used to inactivate pathogens, especially in liquid foods such as fruit juices. Successful eradication of *Listeria innocua* in orange juice by the action of PEF, has been reported and intensely investigated. Other examples include the inactivation of *Listeria monocytogenes* in cherry juice [59].

The mistake often found in the literature is that the electric current flows through the system in addition to the electric field. The voltage applied to uninsulated electrodes immersed in the solution induces redox reactions on the electrodes. Consequently, the electric current flows through the solution with bacteria, significantly contributing to the overall effect.

## 3. Nanotechnology

Nanoparticles synthesized in laboratories are foreign to bacteria and thus evade existing resistance mechanisms [60]. Synthetic nanomaterials are abiological and, therefore, can also circumvent deactivation by resistance mechanisms [61]. Many nanomaterials are used as antimicrobial agents, such as metal-based nanoparticles, carbon-based nanomaterials, polymers, nanocomposites, nanoemulsions, liposomes, and smart nanomaterials [60]. Depending on the nature of interactions between bacterial cells and nanomaterials, these can be considered chemical and physical factors affecting bacteria functioning [62]. Interactions between cells (i.e., cell wall, membranes) and nanoparticles are based on van der Waals forces, electrostatic interactions, hydrogen bonding, and chemical reactions [63]. Possible mechanisms of interaction between nanostructures and cells include uptake of the nanoparticles [62,64,65,66], contact killing [67,68], the effect that arises from released species (e.g., ions) [69,70,71,72,73], and mechanical stress [74,75].

The first step of uptake is the physical interaction between the nanostructure and the cell membrane. This can induce segregation and clustering of nanoobjects on the surface, followed by cell membrane response (lipid segregation, lipid-protein domain formation, the formation of membrane invaginations) [76]. Nanoparticles have little chance to get through the intact bacterial cell wall, where peptidoglycan is present [62]. Only objects of size below a few nanometers can diffuse through the bacterial cell wall [77]. The import of small molecules or peptides can occur, but larger objects need to be degraded via extracellular enzymes (e.g., proteases). Small pieces can then be taken up passively via channels in the membrane or actively via importing pumps [78]. The entry of bigger particles is impossible without previous damage or destabilization of the cell wall (naturally, e.g., during a horizontal transfer of genes or artificially, e.g., due to electroporation or heat shock).

The release of chemical species or release-mediated killing is related to the generation of active species (free ions, radicals) from the nanoparticle surface. Released ions can diffuse inside the cell and block the active center of enzymes, deactivating the protein’s functionality. There are a plenty of recent reviews that focus on the toxic potential of materials at the nanolevel [79,80,81,82,83]. Exposure of the nanomaterial to UV can lead to electron-hole pairs’ activation and, consequently, bond splitting and radical formation. Produced ROS can irreversibly damage cells (e.g., their membrane and cell wall, DNA, and mitochondria), resulting in cell death. Several nanoparticles’ characteristics contribute to ROS generation, which seems to be the best-known paradigm for nanotoxicity [80].

Contact killing or contact-mediated killing refers to the multistep mechanism of killing microbes. There is no consensus on the exact sequence of events [84,85,86], mainly due to the lack of consistency in applied protocols, used microbial strains, and tested experimental conditions. Research suggests that the mechanism is initiated by (i) dissolved ions into the medium that are causing (ii) cell damages, followed by cell membrane rupture. Perforated cell wall allows (iii) ions to diffuse inside and (iv) generate toxic radicals, which lead to further damages and DNA degradation [84]. It should be stressed that most of the data are related to the contact killing effect, referring to copper nanoparticles [84,85,87,88,89,90] and silver nanoparticles [73,86,91,92]. However, reports suggest an alternative contact killing mechanism, namely local destabilization of cell envelope due to interactions with metal atoms. This causes loosening of the envelope structure and rupture of the cell [13].

Mechanical killing is based on the physical killing of cells by mechanical rupture caused by sharp nanostructures [93]. The first example of the nano-based antibacterial surface was discovered at the wings of the clanger cicada. Such a surface comprises nano-sized pillars that trap and kill microbes by pulling bacteria apart upon wings movement [75].

### 3.1. Nanoscale Antibacterial Agents

#### 3.1.1. Nanoparticles

NPs are categorized into organic and inorganic groups [94], hybrid structures, and carbon-based [95]. The inorganic group comprises metal [94], metal oxide [96] nanoparticles, and quantum dots [97]. A comprehensive review on anti-infective applications of metal-oxide nanoparticles was given recently by Abo-Zeid and Williams [98]. Carbon-based nanostructures represent an individual group due to the large variety of structures, namely: graphene, fullerenes, nanotubes, nanofibers, nano-diamond, nanodots, carbon onions, carbon black, and carbon rings. A review of antibacterial properties of carbon-based nanomaterials was published recently by Xin et al. [99].

Organic nano-systems include liposomes, lipid-based nanoparticles, and polymeric nanoparticles. The usage of organic components makes organic nanoparticles perfect candidates for application in controlled drug delivery systems [100,101], bioimaging [102,103], biosensing [104], tissue regeneration [105], and antibacterial applications [106]. In addition to the convenience in non-covalent encapsulation of active substances, such systems also exhibit biodegradability, non-toxicity, stability in circulation in the bloodstream, and the ability to permeate cell membranes effectively [104].

#### 3.1.2. Nanozymes—Nanoparticles Mimicking Enzymes

Metals and non-metals with protein scaffolds (supra-molecular models) are now being used to understand protein folding, optimization, and design. Known for their stability and affordability, these models have been pivotal in discovering new reactivity patterns of enzymes [107]. The models contain a receptor attached to an active site to mimic natural enzymatic reactions [107]. The first metal oxide to be utilized for such a purpose was the iron-oxide-based artificial peroxidase enzyme reported by Gao et al. [108]. Experiments with this enzyme revealed that inorganic nanoparticles could facilitate the oxidation of typical peroxidase substrates and can be used to identify, separate, and detect analytes of choice [109]. The use of nanozymes combines the advantages of both nanoparticles and natural enzymes, such as good stability, controllable size, ease of preparation, multifunctionality, and superior catalytic activity.

Nanozymes might have rough surfaces that increase bacterial cell adhesion and highly irregular edges that function as active sites, exhibiting higher levels of intrinsic peroxidase-like activity [109]. Studies on drug-resistant Gram-negative *E. coli* and Gram-positive *S. aureus* demonstrate the effects of high surface-irregularities on bacterial growth and spurred research towards developing alternative antibiotics [108].

Nanozymes also have an antibacterial function due to their ability to regulate the level of ROS free radicals. Similarly, hydrogen peroxide is used as a disinfectant as it can be decomposed to generate free radicals that attack the major cellular components of bacteria. Nanozymes, along with photocatalytic cooperation, were reported to kill bacteria effectively [110]. Nanozymes, when surface-bound, help in the elimination of antibiotic-resistant bacteria and delay the onset of bacterial resistance emergence. However, when nanozymes are used as coating additives, they enable an inert substrate to inhibit biofilm formation and suppress infection-related immune responses [111].

Nanozymes target the biofilm through the means of reactive oxygen species. ROS, being strong oxidants, can destroy the entire biofilm thoroughly. Other methods include the production of hypohalous acids to destruct the biofilm completely. Nanozymes are often used in combinations for better bactericidal effects [112].

#### 3.1.3. Polymer Nanoparticles

Polymer nanoparticles (PNPs), also called nanospheres or nanocapsules, are prepared either by polymerization of monomers (micro/mini emulsion) or from preformed polymers via solvent evaporation, salting-out, and dialysis [113]. They are defined as antibacterial drug carriers that exhibit physical and chemical stability, are easy to fabricate, and demonstrate easily controllable physicochemical properties, which boost greater targeting efficiency [94].

Certain varieties of PNPs are formed to create lipid-polymer hybrid nanoparticles. One such example, lipid-based surface-functionalized polylactic-co-glycolic acid (PLGA), is both biocompatible and biodegradable. PLGA is also FDA approved for various drug delivery systems and is widely chosen over other nanoparticle types [114]. Antibiotics can also be infused with polymer nanoparticles to increase their activity in multiple ways, such as binding to components around the biofilm, thus increasing the contact time between the bacteria and the drug and protecting the antibiotic from degradation. [114]. Another class of PNPs mimics host defense peptides and finds an application in broad-spectrum treatments [115].

Chitosan nanoparticles (CNPs) also possess antimicrobial activity against a wide range of pathogens. CNPs are prepared by ionic gelation of different concentrations [116]. CNPs loaded with ampicillin, penicillin or diclofenac sodium show increased antibacterial properties [117]. Chitosan nanoparticles display a superior antimicrobial activity against all microorganisms when compared to chitosan and chitin [118]. Additionally, cellulose fibers modified with silver nanoparticles provide good antibacterial properties against *Escherichia coli* and *Staphylococcus aureus* [119]. Contaminated wounds, commonly infected by Gram-positive and Gram-negative bacteria, can be treated by cellulose nanopolymers [120].

Other methods include the conjugation of silver nanoparticles graphene quantum dots’ surface (GQD-AgNP) to target bacteria. Such conjugated nanoparticles have improved the production of reactive oxygen species in light-activable GQDs, and the subsequent transformation of light energy to hyperthermia [121]. Carbon quantum dots can harvest light over a broad spectral range from UV to near-IR [122]. Some of the successful experiments of fabricated silica nanoparticles resulted in a complete and quick eradication of strains such as *E. coli* and *S. aureus* [106]. In addition, nanoparticle systems containing silver have gradually gained recognition in the quest to find the most efficient antimicrobial agent. The broad-spectrum antibacterial properties of silver nanoparticles were also utilized in the case of polymer-Ag nanocomposites synthesized using polysulfone amines as templates [106]. Gold nanoparticles, on the other hand, are primarily used as therapeutic agents when functionalized with polymers. Magnetic nanoparticles have also gained attention over the past decade. A class called super-paramagnetic iron oxide nanoparticles is given preference due to their high biocompatibility and ability to turn off their magnetic properties to remove the external electric field. Poly-rhodamine core-shell nanoparticles are one of the earliest polymer-coated magnetic nanoparticles to be studied for antibacterial applications [106].

#### 3.1.4. Antibacterial Surfaces

Colonization and biofilm formation are initiated by the adhesion of bacterial surfaces [123]. This adhesion leads to a clinical infection resulting in biomaterial failure and chronic infections [11]. Due to the highly resistant nature of biofilms, most antibiotics fail to disrupt them [124]. Hence, novel approaches are adopted to modify surfaces to reduce bacterial adhesion and biofilm formation [125]. One such strategy includes the use of titanium oxide by exploiting its photocatalytic effect resulting in ROS generation [125]. Other methods comprise immobilization of antibacterial substances (e.g., silver) [91], creation of anti-adhesion surfaces [16], and fabrication of structured arrays [126]. Various experiments have proven that nanoparticles can disrupt bacterial membranes and hinder biofilm formation [124,127,128]. For instance, the activity of green synthesized silver NPs was demonstrated on *P. aeruginosa* [123]. *Piper betle* L. (Pb) plant functionalized AgNPs (Pb-AgNPs) significantly decreased the biofilm formation of *P. aeruginosa*. Another example of an antibacterial agent includes the green synthesis of silver nanoparticles using *Berberis vulgaris* leaf and root aqueous extract [129].

Biofilm formation is synergistic with quorum sensing by maintaining cell-to-cell proximity, bacteria can communicate effectively to synchronize gene expression, express virulence, and luminescence via quorum sensing (QS). This enhanced networking greatly favors the spread of biofilm-producing species versus those that do not [124,130,131]. To inhibit QS in vivo, the inhibitory molecule must be of low molecular mass, non-toxic to the eukaryotic hosts where the infection is present, highly specific for the QS-regulators, and chemically stable to reside in the host for a longer duration. Examples of such inhibitors are silver nanoparticles, furanones, QS quenching enzymes, garlic extract, peptides and antibodies, and small molecules acting as enzyme inhibitors [130]. Specific transcription factors control biofilm production and quorum sensing—this has led to the discovery of small-molecule inhibitors (liposome, noisome, PGLA, dendrimers, chitosan) [132] that can modulate morphogenetic conversions and prevent biofilm development [133]. Nanoparticles can help these compounds reach their specific target with a high degree of specificity and accuracy [134]. Some surface-functionalized NPs with β-cyclodextrin (β-CD) or N-acylated homoserine lactonase proteins can prevent the signal molecules from reaching their receptor, thereby turning the system off [124].

### 3.2. Adaptation and Resistance of Bacteria to Nanomaterials

Some of the most common resistance mechanisms to nanoparticles’ antibacterial action involve electrostatic repulsion, ion efflux pumps, expression of extracellular matrices, the adaptation of biofilms, and mutations [96]. Other tolerance mechanisms cause enzyme detoxification, often followed by volatilization [135]. A significant side-effect of such resistance mechanisms to nanoparticles is that they are often accompanied by increased resistance to antibiotics [136]. It was found that nano-resistance began with changes in the shape of bacteria and the modulation in expression of membrane proteins, these changes are reversed when the bacteria are no longer exposed to NPs [137]. In the case of wounds, lung or blood, resistance to metal-based nanoparticles is mediated by biomolecules coronas that reduce NPs binding to pathogens [138].

Although nanoparticles are commonly used in commercial products, there are also worrying reports published on resistance against silver nanoparticles [139,140,141,142]. Recent studies confirm that bacteria can evolve resistance to AgNPs through simple genomic changes such as mutations that regulate heavy metals concentration in the intercellular environment [143]. Silver generally causes isomerization of cis to trans unsaturated membrane fatty acids, leading to increased membrane fluidity. The extent of this isomerization is directly proportional to the toxicity and concentration of membrane affecting agents, i.e., AgNO_3_ and AgNPs [144].

*Enterobacteriaceae* acquire resistance to silver when AgNO_3_ is included in the agar medium used for their culture [145]. This is also confirmed by samples collected from burn sites of patients with infected burns treated with silver sulfadiazine for wound prophylaxis [145]. Silver-resistant mutants have a decreased outer membrane permeability to cephalosporins and are deficient in major porins [146]. Adaptation mechanisms in *Pseudomonas putida* involve the change in the state of unsaturated fatty acids and modification of hydrophobicity of their cell envelopes [144]. Studies on *S. aureus* showed mutations that are protective against nanosilver. These mutations are observed after the removal of silver exposure, denoting heritable characteristics. Thus, silver resistance traits can spread even after silver use is discontinued [147]. However, it is noteworthy that bacteria can only transfer such adaptability to nanoparticles after 114 generations at 0.2 h^−1^ [148]. Endodontic bacteria also show some exciting adaptation mechanisms to silver nanoparticles. These mechanisms are of two types: (a) Intrinsic, which include efflux pumps and downregulation of porins, chromosomal resistance genes, and (b) extrinsic, which include point mutations and plasmids with resistance genes [136]. Studies on resistant clinical bacterial isolates have revealed that such bacteria can reduce ionic silver to elemental silver via reduction, thereby impacting wound management significantly [149]. In other cases, bacteria continue to grow in the presence of AgNPs with an impaired metabolic activity [148].

Bacteria such as *E. coli* develop a reversible resistance towards nanoparticles such as ZnO [137]. Operons also play an important role in metal resistance, some of such metal resistance determinants in bacteria include mer operon for mercury resistance, ars for arsenic resistance, and cad for cadmium resistance [135].

The development of resistance to nanomechanical stress was reported by Matuła et.al. [150]. *E. coli* was exposed to numerous collisions with sharp ZnO nanorods. Nanorods exerted pressure on the cell envelope and punctured them, but at the same time, the cells undergo “healing” [151]. This allowed for adaptation observable in a time scale comparable to the time of acquisition of resistance to antibiotics, i.e., hours [152,153]. It was found that the effect of exposure to physical contact is fixed in phenotype and genome after the removal of the stressing agent.

## 4. Bacteriophages

In the late XIX century, Frederick Twort and Felix d’Herelle were the first to report antibacterial agents with viral-like properties. Bacteriophages, i.e., viruses, that use bacteria as hosts, undergo two major cycles: (i) Lytic, where progeny virions (single phage particles) are created inside the bacterial cell, and their release results in disruption of the host cell, and (ii) lysogenic, where the phage inserts its genetic material inside the bacterial genome and lies dormant.

Temperate phages in the form of prophage (i.e., embedded in the bacterial genome) can be activated, e.g., via external stimuli, and enter the lytic cycle [154]. Phages either cause bacterial lysis via proteins called amurins or they form large pores in the cytoplasmic membrane—through the action of small proteins called holins, which permanently affect the integrity of bacterial cell wall [154,155]. Only in a few cases, especially filamentous phages, can progeny virions be continuously secreted, causing chronic infections [156].

D’Herelle sought to exploit these agents’ therapeutic potential by using them on a boy to cure dysentery. Immediately after this, companies such as L’Oréal and Eli Lilly began preparations for the commercialization of phage therapy. Some institutes also started to be devoted entirely to this aspect of microbiology (e.g., Eliava Institute in Tbilisi).

The world was now set to utilize phages to fight bacterial infections. However, this growth was interrupted by the unreliability of phage therapy in the initial trials and the successful use of penicillin during World War II. This led to a shift of interest towards our present form of medicine. The third millennium showcased an increasing health burden of infections with antibiotic-resistant bacteria, taking the researchers back to where it all began. Phage therapy centers are being set up all over again to overcome MDR using bacteriophages (such as in Hirszfeld Institute) [157].

Several phage-based products curing inner ear infections [158], urinary tract infections [159], typhoid [160], and systemic multi-drug-resistant infections [161] are being tested. The first clinical trial approved by the Food and Drug Administration (FDA) is for intravenous phage therapy in 2019 [162]. Abedon et al. [19] and Cisek et al. [163] gave very compelling reviews on the history of phage therapy, and the current situation is summarized by Altamirano and Barr [164].

Additionally, phage therapy also plays a significant role in veterinary medicine. Mice and chicken infected with *Salmonella* spp. can be treated with *Salmonella* phages with a 90–100% success rate [165]. Other bacteriophage applications include phage-mediated biocontrol, phage bioprocessing for food decontamination, and biodisinfection of objects of veterinary supervision [166]. Certain phage-based products such as ListShield^TM^ are now approved to be used in meat and poultry products against *L. monocytogenes* [167].

### 4.1. Phages Against Bacterial Infections

Despite early success, phage therapies got abandoned with the emergence of antibiotics in the pharmaceutical industry [168]. However, the spread of drug-resistance superbugs and lack of new antibiotics is causing the renaissance of the use of phages against bacteria [169]. Bacteriophages are chosen for this purpose due to their bactericidal effect, low toxicity, high specificity, high rate of replication, easy storage, and lack cross-resistance with antibiotic classes [154]. Since bacteriophages are extremely specific, the patient’s commensal bacteria are not negatively affected during the treatment. Moreover, human cells are not directly affected [170]. There are particular examples of phage internalization by eukaryotic cells. The structure of the *E. coli* receptor is similar to the structure of polysialic acid present on the surface of neuroblastoma cells. Lehti et al. showed that the penetration of eukaryotic cells by *E. coli* phage PK1A2 is possible in vitro [171]. The virus remained in the cells for up to 24 h, but such “infection” did not affect the cells’ viability. Another example showed that the engineered phage could bind and enter the cell, but the replication of M13 was not detected [172].

However, the human immune system reacts to the appearance of the phages in the body. Phages impact immunity directly as they modulate the innate and adaptive immune response through phagocytosis, cytokine response, and antibody production [170]. It is difficult to determine precisely which phage components are responsible for the innate immune response modulation. Studies on induced immune reactions usually were conducted using lysates of phages containing leftovers of lysed bacteria (i.e., membrane proteins, LPS, etc.) [173,174,175].

Humoral response to the phages varies by the type of the virus and type of infection [176]. Immune reaction also depends on the location of bacterial contagion and localization of therapeutic phage injection site [163]. In some instances, antibodies against phages are formed, but in other cases, the human body tends to be non-responsive without developing antibodies [176]. Even small differences in phage coats’ protein composition may affect their circulation time and immunogenicity [177].

The frequency and high level of animal interaction with various types of phages in nature are proved by anti-phage antibodies found in the human sera [163]. Research by Łusiak-Szelachowska et al. has shown that sera of patients with bacterial infections treated with phage cocktails (locally or locally/orally) had high anti-phage activity after fifteen days. Sera of healthy volunteers treated with the same dose of phage therapy showed a low phage inactivation rate [178]. Interestingly, sera of patients who received phages orally did not exhibit high anti-phage activity [178]. On the contrary, Żaczek et al. showed that the majority of 20 patients who received MS1 phage cocktail (orally and/or locally) did not show an increased level of anti-phage antibodies at all. Those studies are of great importance as they rant on human phage therapy effectiveness in humans.

Anti-phage immunoglobulins are one of the most significant factors that may potentially limit the therapeutic effectiveness of phage therapy [179]. Neutralizing antibodies bind viral epitopes within those parts relevant to infecting the bacteria [163]. This limits the potential of using phages as drugs. On the other hand, phages can support inflammatory response against bacteria via lysis of the bacterial cell wall, enabling them to release pathogen-associated molecular patterns (PAMPs) and activate the immune system [180]. Therefore, phage therapies might be very effective since, in addition to direct damaging bacteria cells, phages also activate the human immune system.

Bacteriophages also show antibiofilm activity through depolymerase production. Examples of such phages include the ones isolated from Belgrade wastewaters (ISTD) [181]. Smaller phages specifically encoding (enzyme polymeric substances) EPS-degrading enzymes can penetrate through the biofilms and cause a disturbance [182]. The same was demonstrated in the case of *P. aeruginosa* biofilms in mouse models against cystic fibrosis [182]. Another approach to overcome multi-drug resistance is the use of phages with a combination of antibiotics. A better clearance of bacterial cells with a reduced evolution of phages or antibiotic resistance was observed [183]. The main challenge is to determine the robustness of such an approach, explore the role of immune responses that determine therapeutic outcomes, and establish the phage and antibiotic levels necessary for the therapeutic effect [184].

Supplementation of antibiotic treatment with phages is best suited for cases when the antibiotics do not adequately reach the target area or when the antibiotic resistance is very high. Phage-antibiotic synergy is an evolutionary trade-off wherein bacterial resistance towards phages increases antibiotic susceptibility, resulting in bacterial growth reductions and complete biofilm suppression [185]. Carmen et al. analyzed this relationship by combining a real-time microtiter plate readout with a matrix-like heat map of treatment potencies that measures the synergy between phages and antibiotics, so-called synography. This synography is performed against a drug-resistant group of pathogenic *E. coli* with antibiotic levels ranging from MIC across seven logs of viral load. The results suggest that phages act as adjuvants by lowering the MIC for such strains. Therefore, it is established that lytic phages can rejuvenate an ineffective antibiotic for resistant bacteria [186]. Several new aspects have surfaced with success in antibiotic/phage combination therapy, requiring further study and experimentation. The following questions require answers and guidelines: Is there an optimal ratio of phage particles to the antibiotic molecules, which triggers high clearance levels? Are there still unknown interactions with the host immune system after application of phage/antibiotic treatment that can further our understanding and aid better therapy design? It is also essential to explore the rate at which bacteria develop or lose “immunity” towards phages. Another concern is the accurate identification of the pathogen causing the infection, as phages are extremely specific in their action [187]. Further complications in the application of this therapy, e.g., combination with antibiotics, and in-patient care, also require consideration [188].

Despite the effectiveness of the phage-antibiotic combination, this approach may not be recommended in all cases. For instance, aminoglycoside antibiotics inhibit DNA replication in mycobacteriophage and hence cause interference with pathogen elimination by phages [189].

Another powerful tool against multi-drug resistance is the combination of phages with nanoparticles. Metallic nanoparticles can be functionalized onto phages to support the antibacterial action. While bacteriophages provide the specificity for delivery, the metallic gold particles act as bactericidal agents. Such a combination of phages and nanoparticles is easy to engineer, and the properties of the constituents complement each other in eliminating resistant bacterial infections [96]. Bacteriophages can also be conjugated to gold nanorods. These systems can then be used to kill specific bacterial cells using photothermal ablation, i.e., local generation of heat causing bacterial cell death followed by excitation with near-infrared light [190].

Phages can also be employed to produce novel bio/nanomaterials due to their rapid multiplication with uniform copies. They can either be used as building blocks of such materials or mere templates. Such phage-based nanomaterials have a wide range of applications, their use depends on the properties of individual phages and additional components of the composites [191]. Phages can themselves be used as natural nanoparticles. They can also be engineered to display peptides that have bactericidal effects [192].

### 4.2. Bacterial Adaptations against Phages

#### 4.2.1. Physical Mechanisms

Bacteria can inculcate various changes to limit phage propagation. Under harsh conditions, bacteria resort to the production of structured extracellular polymers, which provides a physical barrier between phages and their receptors [193]. Cells of some marine algae species, *Pseudomonas* and *Azotobacter*, produce alginates that offer an additional protective layer against phages’ attack [77].

Modifications were also found within cell surface receptors’ structure to hinder phage adsorption on the surface [193]. An example of such an adaptation is observed in *S. aureus*, wherein additional cell-wall anchored virulence factors, such as immunoglobulin G-binding protein A, are produced [194]. In consequence, this prevents phage adsorption. As observed in F+ strains of *E. coli*, F plasmid encodes “Trat”—an outer membrane protein employed to modify bacterial cells’ structural conformations, resulting in the inability of phage adsorption [193].

After successful adsorption, phages prepare for DNA injection into the host cell. Superinfection exclusion systems (Sie) act against phage DNA by blocking its entry into the cell. Sie systems comprise membrane-associated proteins that are generally phage-encoded and help protect a lysogenic host from infection by other phages [195].

Other means of protection against bacteriophages include the production of inhibitors that specifically bind to the phage receptors rendering them unable for infection [193]. An exciting example of such an inhibitor is noticed in *E. coli*, wherein the interaction between coliphage and its respective receptor (FhuA) is prevented by the binding of FhuA with an antimicrobial peptide (MccJ25) [196].

#### 4.2.2. Innate Mechanisms

Bacteria use innate mechanisms to protect themselves from their phage predators. The most widely studied is the restriction mechanism (RM). It comprises two enzymes: a restriction endonuclease (REase) and a methyltransferase (MTase) [197]. It is interesting how these two enzymes influence the phage DNA fate such that the unmethylated phage DNA is either recognized by REase and degraded or is methylated by MTase to start the lytic cycle [193]. The REase functions to identify and lyse the foreign DNA sequences, whereas MTase helps recognize self and non-self-DNA via the transfer of methyl groups to the host’s genome. Due to this differentiation ability between self and non-self, RM is considered to function as primitive immune systems [197].

A similar primitive immune mechanism, called bacteriophage exclusion (BREX), is a six-gene cassette including an ATP-dependent protease, an RNA binding protein, a DNA methylase, an alkaline phosphatase domain protein, an ATPase-domain protein, and another protein with unconfirmed function [198]. This system does not affect phage adsorption but inhibits phage replication without degrading phage DNA [199]. BREX provides complete resistance to a broad range of phages by carrying out the host cell’s DNA methylation pattern. This helps in discrimination between host and phage DNA.

The defense island system associated with restriction-modification (DISARM) is also an innate resistance mechanism widespread in bacteria and archaea that protects them against the major families of tailed double-stranded DNA phages. The system is based on the expression of five genes encoding a DNA methylase and four others with a helicase domain, a phospholipase D (PLD) domain, a DUF 1998 domain, and a gene of unknown function [200]. DISARM is a novel multi-gene restriction-modification mechanism that helps the prokaryotes gain resistance against their viruses by marking host DNA to differentiate from that of the pathogen.

The argonaute (ago) family of proteins, generally responsible for RNA interference and silencing, also plays a vital role in providing a barrier against foreign phage DNA. In the case of bacteria such as *Thermus thermophilus*, ago proteins are responsible for cleaving the complementary DNA strands by affecting DNA interference [201]. This is a new addition to the bacterial innate immune system, wherein differentiation between bacterial and phage material is carried out by phosphorothioate (PT) modification on DNA sugar-phosphate backbone [202].

#### 4.2.3. Chemical Defense

Bacteria produce compounds that provide resistance against bacteriophages by intercalating with their DNA and inhibiting replication. There are up to eleven such compounds known so far, out of which daunorubicin, doxorubicin, epirubicin, and idarubicin are used against cancer. These molecules were first found to be produced by *Streptomyces* [203]. Other modes of chemical defense help increase the bacterial cell wall’s permeability for K^+^ ions, thereby preventing genome injection. Examples of such compounds are dequalinium chloride and di-benzimidazole [204].

#### 4.2.4. Abortive Defense

Infected bacterial cells resort to sacrificing themselves to protect the surrounding bacterial population, hence taking one for the team. Such abortive systems (Abi) are generally encoded by mobile genetic elements such as prophages and plasmids.

The best characterized abortive mechanism so far is a two-component rex system comprising rexA and rexB proteins [195]. At the beginning of the infection, phages produce protein-DNA complexes that activate rexA. RexA activates the membrane-anchored rexB, which is an ion channel that causes a drop in membrane potential [193]. This decreases the cellular ATP level and ultimately leads to cell multiplication prevention [193,195]. Due to this, phage infection also aborts as both ATP and ATP-dependent cellular components are now scarce [193].

A type of Abi system, called cyclic oligonucleotide-based anti-phage signaling system (CBASS), is one of the many mechanisms used by bacteria to plan a premature cell death [205]. In the case of phage infection, CBASS leads to the production of signaling cyclic oligonucleotides, which leads to the activation of an effector to promote cell death [205,206].

Other examples involve the action of a serine/threonine kinase (Stk) in *S. epidermidis*. Activated Stk phosphorylates proteins play an essential role in all the major cellular processes such as translation, transcription, metabolism, and repair. Changes in phosphoproteins levels lead to bacterial cell death, thereby preventing the spread of phage infection [207].

HORMA proteins, found in several bacteria such as *E. coli*, also play an essential role in abortive adaptation. These proteins detect phage products and activate a cGAS/DncV-like nucleotidyltransferase to produce cyclic tri-AMP. This second messenger causes cleavage of ds DNA and subsequent destruction of cells [207].

*Lactococcus lactis* shows various Abi systems due to a range of predators attacking it [195]. Thus, Abi systems form a resistance mechanism limiting phage replication in a bacterial population by promoting cell death.

### 4.3. Bacterial Resistance Mechanisms

Temperate bacteriophages form prophage by inserting their genome into bacterial cells. Some DNA fragments are left behind that help surviving bacteria acquire immunity (from bacteriophages) during subsequent infections. These fragments might also be horizontally transferred to other bacterial cells, providing them with similar immunity. Such DNA sequences family has come to be known as clustered regularly short palindromic repeats (CRISPR).

CRISPR has an associated protein, an endonuclease responsible for creating cuts in a double-stranded DNA, thereby helping modify the genome. This protein is called Cas9, and its association with a guide RNA, responsible for matching the target gene, is called the CRISPR-Cas9 system. Cas protein is directed by RNA-spacers (flanked by repeats) to target DNA and cleave it [146]. These spacers are fragments of DNA congregated from phages that have attacked the bacterial cell in the cell. Insertion of spacers into CRISPR loci on the host genome ultimately leads to the prevention against phage infection [208]. A popular application of CRISPR-Cas9 is in the treatment of infectious diseases such as HIV [145]. Other applications include the target and cleavage of DNA responsible for antibiotic resistance [146].

It is speculated that the CRISPR-Cas immune system of certain bacteria such as *P. aeruginosa* is controlled by quorum sensing [209]. The CRISPR-Cas9 system protects bacteria from horizontally transferred mobile elements. MDR bacteria, lacking this system, acquire new genes easily and rapidly adapt to new antibiotics [9]. On the other hand, CRISPR-Cas9 prevents the completion of the phage life cycle.

In the context of the ever-evolving arms race between bacteria and phages, it was now crucial for the latter to develop a system of their own against CRISPR. Such a system, called anti CRISPR, was soon discovered within bacteriophages. Anti-CRISPR (Acr) proteins that can easily block different CRISPR-Cas systems were found in the genomes of viruses, bacteria, and archaea. Acr genes were first discovered in *P. aeruginosa* where they encode a range of small proteins preventing the functioning of the CRISPR-Cas9 system against the bacterial genome [210]. Acr-phages overcome CRISPR with the first phage blocking the host CRISPR-Cas immune system to allow a subsequent Acr-phage to attack [211]. Acr-phages work as a community to replicate and escape extinction successfully [212].

Acr proteins specifically inhibit the CRISPR-Cas system, and therefore have an enormous potential for application as modulators of genome editing tools. Several approaches were employed to discover Acr families, two primary ones being: (i) Guilt by association and (ii) self-targeting. Guilt by association functions by searching for helix-turn-helix (HTH)-containing proteins encoded downstream of Acr proteins. Such proteins are referred to as Aca (anti-CRISPR associated) and are more conserved than Acr themselves. Self-targeting is referred to CRISPR-Cas systems that enclose spacers targeting regions of the same genome. Organisms with such self-targeting genomes can only survive with the presence of Acrs to prevent CRISPR-Cas from functioning [213].

Anti-CRISPR proteins have also found application in precise and efficient gene editing. One such example is the anti-CRISPR protein, AcrIIA4, fused with the N-terminal region of human Cdt1 that is degraded in S and G_2_ phases of the cell cycle. The expression of AcrIIA4-Cdt1 can increase the frequency of homology-directed repair (HDR) in these phases. This efficiency can also be enhanced by tuning the delivery timing of SpyCas9-single guide RNA (sgRNA) ribonucleoprotein (RNP) complexes. This combination of SpyCas9 and AcrIIA4-Cdt1 is the cell cycle-dependent Cas9 activation system for successful genome editing [214].

Acr genes are found adjacent to genes encoding the HTH DNA-binding motif. These HTH encoding genes are used as markers to identify anti-CRISPR families. Interestingly, another system, popularly known as the anti-CRISPR system, comprises these HTH encoding genes. Their function is to act as the repressor of Acr promoter, thereby attenuating CRISPR transcription. Not much is known about such systems yet, but they have given a new direction to CRISPR-based genome-editing tools [215].

To summarize, bacteria counteract phage attack by preventing phage adsorption through biofilms, inhibiting DNA injection via inactivation of proteins involved in the cell wall synthesis, targeting bacteriophage nucleic acids with the help of nucleases, and employing CRISPR systems against the attacking bacteriophage. Another ingenious method, known as abortive infection, is an altruistic action wherein the release of functional phage virions is prevented by the host cell’s programmed cell death [216].

## 5. Discussion and Future Perspectives

Many new approaches are employed to fight antibiotic resistance in bacteria. Many of these include a better delivery system for antibiotics that ensure a greater diffusion at the target site via specific delivery systems. Scientists have also explored bacterial machinery to use it against them in several ways. Quorum sensing and biofilm targeting are two such approaches that have gained attention and produced successful results. Several antibacterial agents are explored to target infectious bacteria that are now heavily resistant to different antibiotics classes. The field of nanomedicine is growing every day to fight the rapid evolution of superbugs. Metal and metal oxide NPs, polymer nanoparticles, nanozymes, and phage mimicking nanoparticles are the main components of this treatment mode.

Bacteriophages continue to surprise researchers with their antibacterial properties and their specificity towards their host. This helps ensure the safety of the human microbiome while eradicating pathological bacterial species. Phages are also modified to attack bacteria, either in combination with antibiotics or with nanoparticles.

Phage therapies were mostly abandoned when antibiotics were discovered and developed. This was primarily because antibiotics were very efficient against a much wider range of bacterial infections. In the case of phage therapies, each bacteria species requires a separate phage to be administered. There are also specific requirements for the formulation, storage, and administration of phages [217]. The main advantage of phages, and the cause of the renaissance of phage therapies, is related to the appearance of superbugs. While it is easier to gain resistance against antibiotics, bacteriophage-resistant bacteria are still scarce. Phages undergo evolution, and thus there is a constant arms race between bacteria and phages. Additionally, bacteriophages are harmless to any other cells apart from their host. At the same time, antibiotics can produce side effects by interacting with human cells’ regular functioning. Their wide range of action also affects the bacteria required for the normal functioning of the body. Several reports of bacteriophages and antibiotics’ combined effect showed a greater success rate than either of them used individually. Furthermore, nanoparticles can also be used as both antibiotics and as vehicles for carrying antibiotics for a site-specific treatment. NPs also show antimicrobial activity by targeting biofilms.

It is intriguing how bacteria have found ways to escape these treatment methodologies by developing resistance against physical/mechanical factors, the action of nanostructures, and the attack of bacteriophages. CRISPR systems and their possible application in combating MDR are very nascent and have yet to further be explored.

The main message for the researchers working on novel antibacterial agents is to include studies on the sustainability of the newly developed methods and explore new combination treatments. It is not enough to report the antibacterial action, but it is crucial to create a means to verify if and how fast bacteria can develop resistance. It is imperative in the case of agents, which utilize very general mechanisms to fight bacteria. Regrettably, the appearance of resistance ultimately renders such antibacterial agents obsolete.

## Data Availability

The datasets used and/or analyzed during the current study are available from the corresponding author upon reasonable request.

## References

[1] Pelfrene E., Botgros R., Cavaleri M. (2021). Antimicrobial multidrug resistance in the era of COVID-19: A forgotten plight?. Antimicrob. Resist. Infect. Control.

[2] Capita R., Alonso-Calleja C. (2013). Antibiotic-Resistant Bacteria: A Challenge for the Food Industry. Crit. Rev. Food Sci. Nutr..

[3] Nji E., Kazibwe J., Hambridge T., Joko C.A., Larbi A.A., Damptey L.A.O., Nkansa-Gyamfi N.A., Stålsby Lundborg C., Lien L.T.Q. (2021). High prevalence of antibiotic resistance in commensal Escherichia coli from healthy human sources in community settings. Sci. Rep..

[4] Neill J.O. (2014). Antimicrobial Resistance: Tackling a Crisis for the Health and Wealth of Nations. Review on Antimicrobial Resistance Chaired.

[5] Smith M. (2017). Antibiotic Resistance Mechanisms. Journeys Med. Res. Three Cont. Over.

[6] Ventola C.L. (2015). The antibiotic resistance crisis: Part 1: Causes and threats. Pharm. Ther..

[7] Wang W., Arshad M.I., Khurshid M., Rasool M.H., Nisar M.A., Aslam M.A., Qamar M.U. (2018). Antibiotic resistance: A rundown of a global crisis. Infect. Drug Resist..

[8] Liu Y.-Y., Wang Y., Walsh T.R., Yi L.-X., Zhang R., Spencer J., Doi Y., Tian G., Dong B., Huang X. (2015). Emergence of plasmid-mediated colistin resistance mechanism MCR-1 in animals and human beings in China: A microbiological and molecular biological study. Lancet Infect. Dis..

[9] Wang R., Van Dorp L., Shaw L.P., Bradley P., Wang Q., Wang X., Jin L., Zhang Q., Liu Y., Rieux A. (2018). The global distribution and spread of the mobilized colistin resistance gene mcr-1. Nat. Commun..

[10] Otto C., Zahn S., Rost F., Zahn P., Jaros D., Rohm H. (2011). Physical Methods for Cleaning and Disinfection of Surfaces. Food Eng. Rev..

[11] Theuretzbacher U., Piddock L.J.V. (2019). Non-traditional Antibacterial Therapeutic Options and Challenges. Cell Host Microbe.

[12] Beyth N., Houri-Haddad Y., Domb A., Khan W., Hazan R. (2015). Alternative antimicrobial approach: Nano-antimicrobial materials. Evid. Based Complement. Altern. Med..

[13] Wybrańska K., Paczesny J., Serejko K., Sura K., Włodyga K., Dzięcielewski I., Jones S.T., Sliwa A., Wybrańska I., Hołyst R. (2015). Gold-oxoborate nanocomposites and their biomedical applications. ACS Appl. Mater. Interfaces.

[14] Tripathy A., Sen P., Su B., Briscoe W.H. (2017). Natural and bioinspired nanostructured bactericidal surfaces. Adv. Colloid Interface Sci..

[15] Pillai P.P., Kowalczyk B., Kandere-Grzybowska K., Borkowska M., Grzybowski B.A. (2016). Engineering Gram Selectivity of Mixed-Charge Gold Nanoparticles by Tuning the Balance of Surface Charges. Angew. Chemie Int. Ed..

[16] Vallet-Regí M., González B., Izquierdo-Barba I. (2019). Nanomaterials as promising alternative in the infection treatment. Int. J. Mol. Sci..

[17] Sulakvelidze A., Alavidze Z.J., Glenn Morris J. (2001). Bacteriophage Therapy. Antimicrob. Agents Chemother..

[18] Parfitt T. (2005). Georgia: An unlikely stronghold for bacteriophage therapy. Lancet.

[19] Abedon S.T., Kuhl S.J., Blasdel B.G., Kutter E.M. (2011). Phage treatment of human infections. Bacteriophage.

[20] McCallin S., Alam Sarker S., Barretto C., Sultana S., Berger B., Huq S., Krause L., Bibiloni R., Schmitt B., Reuteler G. (2013). Safety analysis of a Russian phage cocktail: From MetaGenomic analysis to oral application in healthy human subjects. Virology.

[21] Novickij V., Stanevičiene R., Vepštaite-Monstaviče I., Gruškiene R., Krivorotova T., Sereikaite J., Novickij J., Serviene E. (2018). Overcoming antimicrobial resistance in bacteria using bioactive magnetic nanoparticles and pulsed electromagnetic fields. Front. Microbiol..

[22] Sun W., Qian X., Gu J., Wang X.J., Duan M.L. (2016). Mechanism and Effect of Temperature on Variations in Antibiotic Resistance Genes during Anaerobic Digestion of Dairy Manure. Sci. Rep..

[23] Li H., Gänzle M. (2016). Some like it hot: Heat resistance of Escherichia coli in food. Front. Microbiol..

[24] Loraine G., Chahine G., Hsiao C.T., Choi J.K., Aley P. (2012). Disinfection of gram-negative and gram-positive bacteria using DynaJets^®^ hydrodynamic cavitating jets. Ultrason. Sonochem..

[25] Mai-Prochnow A., Clauson M., Hong J., Murphy A.B. (2016). Gram positive and Gram negative bacteria differ in their sensitivity to cold plasma. Sci. Rep..

[26] Cebrián G., Condón S., Mañas P. (2017). Physiology of the Inactivation of Vegetative Bacteria by Thermal Treatments: Mode of Action, Influence of Environmental Factors and Inactivation Kinetics. Foods.

[27] Yoon Y., Lee H., Lee S., Kim S., Choi K.H. (2015). Membrane fluidity-related adaptive response mechanisms of foodborne bacterial pathogens under environmental stresses. Food Res. Int..

[28] Cebrián G., Mañas P., Condón S. (2016). Comparative resistance of bacterial foodborne pathogens to non-thermal technologies for food preservation. Front. Microbiol..

[29] René O., Alix J.H. (2011). Late steps of ribosome assembly in E. coli are sensitive to a severe heat stress but are assisted by the HSP70 chaperone machine. Nucleic Acids Res..

[30] Guyot S., Pottier L., Ferret E., Gal L., Gervais P. (2010). Physiological responses of Escherichia coli exposed to different heat-stress kinetics. Arch. Microbiol..

[31] Szeto W., Yam W.C., Huang H., Leung D.Y.C. (2020). The efficacy of vacuum-ultraviolet light disinfection of some common environmental pathogens. BMC Infect. Dis..

[32] Maclean M., MacGregor S.J., Anderson J.G., Woolsey G. (2009). Inactivation of bacterial pathogens following exposure to light from a 405-nanometer light-emitting diode array. Appl. Environ. Microbiol..

[33] Kovach C.R., Taneli Y., Neiman T., Dyer E.M., Arzaga A.J.A., Kelber S.T. (2017). Evaluation of an ultraviolet room disinfection protocol to decrease nursing home microbial burden, infection and hospitalization rates. BMC Infect. Dis..

[34] Silva J., Castillo G., Callejas L., López H., Olmos J. (2006). Frequency of transferable multiple antibiotic resistance amongst coliform bacteria isolated from a treated sewage effluent in Antofagasta, Chile. Electron. J. Biotechnol..

[35] Lamprecht-Grandío M., Cortesão M., Mirete S., de la Cámara M.B., de Figueras C.G., Pérez-Pantoja D., White J.J., Farías M.E., Rosselló-Móra R., González-Pastor J.E. (2020). Novel Genes Involved in Resistance to both Ultraviolet Radiation and Perchlorate from the Metagenomes of Hypersaline Environments. Front. Microbiol..

[36] Mofidi A.A., Rochelle P.A., Chou C.I., Mehta H.M., Verne L., Linden K.G. (2002). Bacterial Survival After Ultraviolet Light Disinfection: Resistance, Regrowth and Repair. Am. Water Work. Assoc. Annu. Conf. Exhib..

[37] Pérez V., Hengst M., Kurte L., Dorador C., Jeffrey W.H., Wattiez R., Molina V., Matallana-Surget S. (2017). Bacterial survival under extreme UV radiation: A comparative proteomics study of Rhodobacter sp., isolated from high altitude wetlands in Chile. Front. Microbiol..

[38] Alcántara-Díaz D., Breña-Valle M., Serment-Guerrero J. (2004). Divergent adaptation of Escherichia coli to cyclic ultraviolet light exposures. Mutagenesis.

[39] Marizcurrena J.J., Morel M.A., Braña V., Morales D., Martinez-López W., Castro-Sowinski S. (2017). Searching for novel photolyases in UVC-resistant Antarctic bacteria. Extremophiles.

[40] Monsalves M.T., Ollivet-Besson G.P., Amenabar M.J., Blamey J.M. (2020). Isolation of a psychrotolerant and UV-C-resistant bacterium from elephant island, antarctica with a highly thermoactive and thermostable catalase. Microorganisms.

[41] Díaz-Riaño J., Posada L., Acosta I.C., Ruíz-Pérez C., García-Castillo C., Reyes A., Zambrano M.M. (2019). Computational search for UV radiation resistance strategies in Deinococcus swuensis isolated from Paramo ecosystems. PLoS ONE.

[42] Moreirinha C., Almeida A., Saraiva J.A., Delgadillo I. (2016). High-pressure processing effects on foodborne bacteria by mid-infrared spectroscopy analysis. LWT Food Sci. Technol..

[43] Chikuma S., Kasahara R., Kato C., Tamegai H. (2007). Bacterial adaptation to high pressure: A respiratory system in the deep-sea bacterium Shewanella violacea DSS12. FEMS Microbiol. Lett..

[44] Masson P., Tonello C., Balny C. (2001). High-pressure biotechnology in medicine and pharmaceutical science. J. Biomed. Biotechnol..

[45] Yoo J.H. (2018). Review of disinfection and sterilization—Back to the basics. Infect. Chemother..

[46] Reineke K., Mathys A., Knorr D. (2011). The impact of high pressure and temperature on bacterial spores: Inactivation mechanisms of Bacillus subtilis above 500 MPa. J. Food Sci..

[47] Vanlint D., Rutten N., Michiels C.W., Aertsen A. (2012). Emergence and stability of high-pressure resistance in different food-borne pathogens. Appl. Environ. Microbiol..

[48] Mota M.J., Lopes R.P., Delgadillo I., Saraiva J.A. (2013). Microorganisms under high pressure—Adaptation, growth and biotechnological potential. Biotechnol. Adv..

[49] Pudasaini S., Perera A.T.K., Ng S.H., Yang C. (2021). Bacterial inactivation via microfluidic electroporation device with insulating micropillars. Electrophoresis.

[50] Kotnik T., Frey W., Sack M., Haberl Meglič S., Peterka M., Miklavčič D. (2015). Electroporation-based applications in biotechnology. Trends Biotechnol..

[51] Martens S.L., Klein S., Barnes R.A., TrejoSanchez P., Roth C.C., Ibey B.L. (2020). 600-ns pulsed electric fields affect inactivation and antibiotic susceptibilities of Escherichia coli and Lactobacillus acidophilus. AMB Express.

[52] Escoffre J.M., Portet T., Wasungu L., Teissié J., Dean D., Rols M.P. (2009). What is (Still not) known of the mechanism by which electroporation mediates gene transfer and expression in cells and tissues. Mol. Biotechnol..

[53] Garcia P.A., Ge Z., Moran J.L., Buie C.R. (2016). Microfluidic screening of electric fields for electroporation. Sci. Rep..

[54] Cannon R., Ellis S., Hayes D., Narayanan G., Martin R.C.G. (2013). Safety and early efficacy of irreversible electroporation for hepatic tumors in proximity to vital structures. J. Surg. Oncol..

[55] Pillet F., Formosa-Dague C., Baaziz H., Dague E., Rols M.P. (2016). Cell wall as a target for bacteria inactivation by pulsed electric fields. Sci. Rep..

[56] Costello S.R. (2012). Effect of Electric Field on Growth Kinetics of Yogurt Starter Cultures, Lactobacillus Bulgaricus and Streptococcus Thermophilus. Master’s Thesis.

[57] Gall I., Herzberg M., Oren Y. (2013). The effect of electric fields on bacterial attachment to conductive surfaces. Soft Matter.

[58] Yonemoto Y., Yamashita T., Muraji M., Tatebe W., Ooshima H., Kato J., Kimura A., Murata K. (1993). Resistance of yeast and bacterial spores to high voltage electric pulses. J. Ferment. Bioeng..

[59] Puligundla P., Pyun Y.R., Mok C. (2018). Pulsed electric field (PEF) technology for microbial inactivation in low-alcohol red wine. Food Sci. Biotechnol..

[60] Makabenta J.M.V., Nabawy A., Li C.H., Schmidt-Malan S., Patel R., Rotello V.M. (2020). Nanomaterial-based therapeutics for antibiotic-resistant bacterial infections. Nat. Rev. Microbiol..

[61] Pelgrift R.Y., Friedman A.J. (2013). Nanotechnology as a therapeutic tool to combat microbial resistance. Adv. Drug Deliv. Rev..

[62] Stark W.J. (2011). Nanoparticles in biological systems. Angew. Chemie Int. Ed..

[63] Doane T., Burda C. (2013). Nanoparticle mediated non-covalent drug delivery. Adv. Drug Deliv. Rev..

[64] Limbach L.K., Li Y., Grass R.N., Brunner T.J., Hintermann M.A., Muller M., Gunther D., Stark W.J. (2005). Oxide Nanoparticle Uptake in Human Lung Fibroblasts: Effects of Particle Size, Agglomeration, and Diffusion at Low Concentrations. Environ. Sci. Technol..

[65] Studer A.M., Limbach L.K., Van Duc L., Krumeich F., Athanassiou E.K., Gerber L.C., Moch H., Stark W.J. (2010). Nanoparticle cytotoxicity depends on intracellular solubility: Comparison of stabilized copper metal and degradable copper oxide nanoparticles. Toxicol. Lett..

[66] Treuel L., Jiang X., Nienhaus G.U. (2013). New views on cellular uptake and trafficking of manufactured nanoparticles. J. R. Soc. Interface.

[67] Ji Z., Wang X., Zhang H., Lin S., Meng H., Sun B., George S., Xia T., Nel A.E., Zink J.I. (2012). Designed synthesis of CeO_2_ nanorods and nanowires for studying toxicological effects of high aspect ratio nanomaterials. ACS Nano.

[68] Jacob S.J.P., Mohammed H., Murali K., Kamarudeen M. (2012). Synthesis of silver nanorods using Coscinium fenestratum extracts and its cytotoxic activity against Hep-2 cell line. Colloids Surf. B. Biointerfaces.

[69] Rai M.K., Deshmukh S.D., Ingle A.P., Gade A.K. (2012). Silver nanoparticles: The powerful nanoweapon against multidrug-resistant bacteria. J. Appl. Microbiol..

[70] Haider A., Kang I.-K. (2015). Preparation of Silver Nanoparticles and Their Industrial and Biomedical Applications: A Comprehensive Review. Adv. Mater. Sci. Eng..

[71] Rauwel P., Rauwel E., Ferdov S., Singh M.P. (2015). Silver Nanoparticles: Synthesis, Properties, and Applications. Adv. Mater. Sci. Eng..

[72] Tubert-Brohman I., Sherman W., Repasky M., Beuming T. (2013). Improved Docking of Polypeptides with Glide. J. Chem. Inf. Model..

[73] Li Y., Dong Y., Yang Y., Yu P., Zhang Y., Hu J., Li T., Zhang X., Liu X., Xu Q. (2019). Rational Design of Silver Gradient for Studying Size Effect of Silver Nanoparticles on Contact Killing. ACS Biomater. Sci. Eng..

[74] Ivanova E.P., Hasan J., Webb H.K., Truong V.K., Watson G.S., Watson J.A., Baulin V.A., Pogodin S., Wang J.Y., Tobin M.J. (2012). Natural bactericidal surfaces: Mechanical rupture of pseudomonas aeruginosa cells by cicada wings. Small.

[75] Pogodin S., Hasan J., Baulin V.A., Webb H.K., Truong V.K., Phong Nguyen T.H., Boshkovikj V., Fluke C.J., Watson G.S., Watson J.A. (2013). Biophysical model of bacterial cell interactions with nanopatterned cicada wing surfaces. Biophys. J..

[76] Johannes L., Mayor S. (2010). Induced domain formation in endocytic invagination, lipid sorting, and scission. Cell.

[77] Silhavy T.J., Kahne D., Walker S. (2010). The Bacterial Cell Envelope. Cold Spring Harb Perspect. Biol..

[78] Fuerst J.A., Sagulenko E. (2010). Protein uptake by bacteria. Commun. Integr. Biol..

[79] Hajipour M.J., Fromm K.M., Akbar Ashkarran A., de Jimenez Aberasturi D., de Larramendi I.R., Rojo T., Serpooshan V., Parak W.J., Mahmoudi M. (2012). Antibacterial properties of nanoparticles. Trends Biotechnol..

[80] Nel A., Xia T., Mädler L., Li N. (2006). Toxic potential of materials at the nanolevel. Science.

[81] Xia Q., Hwang H.-M., Ray P.C., Yu H. (2014). Mechanisms of nanotoxicity: Generation of reactive oxygen species. J. Food Drug Anal..

[82] Walters C., Pool E., Somerset V. (2016). Nanotoxicology: A Review. Toxicology—New Aspects to This Scientific Conundrum.

[83] Lewinski N., Colvin V., Drezek R. (2008). Cytotoxicity of Nanoparticles. Small.

[84] Vincent M., Duval R.E., Hartemann P., Engels-Deutsch M. (2018). Contact killing and antimicrobial properties of copper. J. Appl. Microbiol..

[85] Mathews S., Hans M., Mücklich F., Solioz M. (2013). Contact killing of bacteria on copper is suppressed if bacterial-metal contact is prevented and is induced on iron by copper ions. Appl. Environ. Microbiol..

[86] Sondi I., Salopek-Sondi B. (2004). Silver nanoparticles as antimicrobial agent: A case study on E. coli as a model for Gram-negative bacteria. J. Colloid Interface Sci..

[87] Hans M., Mathews S., Mücklich F., Solioz M. (2016). Physicochemical properties of copper important for its antibacterial activity and development of a unified model. Biointerphases.

[88] Vincent M., Hartemann P., Engels-Deutsch M. (2016). Antimicrobial applications of copper. Int. J. Hyg. Environ. Health.

[89] Slavin Y.N., Asnis J., Häfeli U.O., Bach H. (2017). Metal nanoparticles: Understanding the mechanisms behind antibacterial activity. J. Nanobiotechnol..

[90] Tao B., Lin C., Deng Y., Yuan Z., Shen X., Chen M., He Y., Peng Z., Hu Y., Cai K. (2019). Copper-nanoparticle-embedded hydrogel for killing bacteria and promoting wound healing with photothermal therapy. J. Mater. Chem. B.

[91] Agnihotri S., Mukherji S., Mukherji S. (2013). Immobilized silver nanoparticles enhance contact killing and show highest efficacy: Elucidation of the mechanism of bactericidal action of silver. Nanoscale.

[92] Li Z., Lee D., Sheng X., Cohen R.E., Rubner M.F. (2006). Two-Level Antibacterial Coating with Both Release-Killing and Contact-Killing Capabilities. Langmuir.

[93] Linklater D.P., Baulin V.A., Juodkazis S., Crawford R.J., Stoodley P., Ivanova E.P. (2021). Mechano-bactericidal actions of nanostructured surfaces. Nat. Rev. Microbiol..

[94] Eleraky N.E., Allam A., Hassan S.B., Omar M.M. (2020). Nanomedicine fight against antibacterial resistance: An overview of the recent pharmaceutical innovations. Pharmaceutics.

[95] Horikoshi S., Serpone N. (2013). Microwaves in Nanoparticle Synthesis: Fundamentals and Applications.

[96] Salas Orozco M.F., Niño-Martínez N., Martínez-Castañón G.A., Méndez F.T., Ruiz F. (2019). Molecular mechanisms of bacterial resistance to metal and metal oxide nanoparticles. Int. J. Mol. Sci..

[97] Reyes-Esparza J., Martínez-Mena A., Gutiérrez-Sancha I., Rodríguez-Fragoso P., Cruz G.G., Mondragón R., Rodríguez-Fragoso L. (2015). Synthesis, characterization and biocompatibility of cadmium sulfide nanoparticles capped with dextrin for in vivo and in vitro imaging application. J. Nanobiotechnology.

[98] Abo-Zeid Y., Williams G.R. (2020). The potential anti-infective applications of metal oxide nanoparticles: A systematic review. Wiley Interdiscip. Rev. Nanomed. Nanobiotechnology.

[99] Xin Q., Shah H., Nawaz A., Xie W., Akram M.Z., Batool A., Tian L., Jan S.U., Boddula R., Guo B. (2019). Antibacterial Carbon-Based Nanomaterials. Adv. Mater..

[100] Kakkar A., Traverso G., Farokhzad O.C., Weissleder R., Langer R. (2017). Evolution of macromolecular complexity in drug delivery systems. Nat. Rev. Chem..

[101] Ishihara K., Chen W., Liu Y., Tsukamoto Y., Inoue Y. (2016). Cytocompatible and multifunctional polymeric nanoparticles for transportation of bioactive molecules into and within cells. Sci. Technol. Adv. Mater..

[102] Miao Q., Xie C., Zhen X., Lyu Y., Duan H., Liu X., Jokerst J.V., Pu K. (2017). Molecular afterglow imaging with bright, biodegradable polymer nanoparticles. Nat. Biotechnol..

[103] Pu K., Chattopadhyay N., Rao J. (2016). Recent advances of semiconducting polymer nanoparticles in in vivo molecular imaging. J. Control. Release.

[104] Kairdolf B.A., Qian X., Nie S. (2017). Bioconjugated Nanoparticles for Biosensing, In Vivo Imaging, and Medical Diagnostics. Anal. Chem..

[105] Van Rijt S., Habibovic P. (2017). Enhancing regenerative approaches with nanoparticles. J. R. Soc. Interface.

[106] Lam S.J., Wong E.H.H., Boyer C., Qiao G.G. (2018). Antimicrobial polymeric nanoparticles. Prog. Polym. Sci..

[107] Korschelt K., Tahir M.N., Tremel W. (2018). A Step into the Future: Applications of Nanoparticle Enzyme Mimics. Chemistry.

[108] Cao F., Zhang L., Wang H., You Y., Wang Y., Gao N., Ren J., Qu X. (2019). Defect-Rich Adhesive Nanozymes as Efficient Antibiotics for Enhanced Bacterial Inhibition. Angew. Chemie Int. Ed..

[109] Singh S. (2019). Nanomaterials exhibiting enzyme-like properties (Nanozymes): Current advances and future perspectives. Front. Chem..

[110] Meng Y., Li W., Pan X., Gadd G.M. (2020). Applications of nanozymes in the environment. Environ. Sci. Nano.

[111] Gao F., Shao T., Yu Y., Xiong Y., Yang L. (2021). Surface-bound reactive oxygen species generating nanozymes for selective antibacterial action. Nat. Commun..

[112] Yang D., Chen Z., Gao Z., Tammina S.K., Yang Y. (2020). Nanozymes used for antimicrobials and their applications. Colloids Surfaces B Biointerfaces.

[113] Rao J.P., Geckeler K.E. (2011). Polymer nanoparticles: Preparation techniques and size-control parameters. Prog. Polym. Sci..

[114] Forier K., Raemdonck K., De Smedt S.C., Demeester J., Coenye T., Braeckmans K. (2014). Lipid and polymer nanoparticles for drug delivery to bacterial biofilms. J. Control. Release.

[115] Gupta A., Landis R.F., Li C.H., Schnurr M., Das R., Lee Y.W., Yazdani M., Liu Y., Kozlova A., Rotello V.M. (2018). Engineered Polymer Nanoparticles with Unprecedented Antimicrobial Efficacy and Therapeutic Indices against Multidrug-Resistant Bacteria and Biofilms. J. Am. Chem. Soc..

[116] Alqahtani F.Y., Aleanizy F.S., El Tahir E., Alhabib H., Alsaif R., Shazly G., Alqahtani H., Alsarra I.A., Mahdavi J. (2020). Antibacterial activity of chitosan nanoparticles against pathogenic N. gonorrhoea. Int. J. Nanomed..

[117] Alqahtani F.Y., Aleanizy F.S., El Tahir E., Alquadeib B.T., Alsarra I.A., Alanazi J.S., Abdelhady H.G. (2019). Preparation, characterization, and antibacterial activity of diclofenac-loaded chitosan nanoparticles. Saudi Pharm. J..

[118] Divya K., Vijayan S., George T.K., Jisha M.S. (2017). Antimicrobial properties of chitosan nanoparticles: Mode of action and factors affecting activity. Fibers Polym..

[119] Smiechowicz E., Niekraszewicz B., Kulpinski P., Dzitko K. (2018). Antibacterial composite cellulose fibers modified with silver nanoparticles and nanosilica. Cellulose.

[120] Barud H.S., Regiani T., Marques R.F.C., Lustri W.R., Messaddeq Y., Ribeiro S.J.L. (2011). Antimicrobial bacterial cellulose-silver nanoparticles composite membranes. J. Nanomater..

[121] Mei L., Cao F., Zhang L., Xu J., Xu Z., Yu Y., Zhang X., Shi Y., Li X., Cheng K. (2020). Ag-Conjugated graphene quantum dots with blue light-enhanced singlet oxygen generation for ternary-mode highly-efficient antimicrobial therapy. J. Mater. Chem. B.

[122] Dong X., Liang W., Meziani M.J., Sun Y.P., Yang L. (2020). Carbon dots as potent antimicrobial agents. Theranostics.

[123] Shah S., Gaikwad S., Nagar S., Kulshrestha S., Vaidya V., Nawani N., Pawar S. (2019). Biofilm inhibition and anti-quorum sensing activity of phytosynthesized silver nanoparticles against the nosocomial pathogen Pseudomonas aeruginosa. Biofouling.

[124] Baptista P.V., McCusker M.P., Carvalho A., Ferreira D.A., Mohan N.M., Martins M., Fernandes A.R. (2018). Nano-strategies to fight multidrug resistant bacteria—“A Battle of the Titans”. Front. Microbiol..

[125] Arango-Santander S., Pelaez-Vargas A., Freitas S.C., García C. (2018). A novel approach to create an antibacterial surface using titanium dioxide and a combination of dip-pen nanolithography and soft lithography. Sci. Rep..

[126] Hussain Bhat A., Khan I., Jawaid M., Suliman F.O., Al-Lawati H., Muhamed S., Editors A.-K. Advanced Structured Materials. Nanomaterials for Healthcare, Energy and Environment.

[127] Annunziato G. (2019). Strategies to overcome antimicrobial resistance (AMR) making use of non-essential target inhibitors: A review. Int. J. Mol. Sci..

[128] Díaz C., Schilardi P.L., Salvarezza R.C., De Mele M.F.L. (2007). Nano/microscale order affects the early stages of biofilm formation on metal surfaces. Langmuir.

[129] Behravan M., Hossein Panahi A., Naghizadeh A., Ziaee M., Mahdavi R., Mirzapour A. (2019). Facile green synthesis of silver nanoparticles using Berberis vulgaris leaf and root aqueous extract and its antibacterial activity. Int. J. Biol. Macromol..

[130] Bhardwaj K.A., Vinothkumar K., Rajpara N. (2013). Bacterial Quorum Sensing Inhibitors: Attractive Alternatives for Control of Infectious Pathogens Showing Multiple Drug Resistance. Recent Pat. Antiinfect. Drug Discov..

[131] Rutherford S.T., Bassler B.L. (2012). Bacterial quorum sensing: Its role in virulence and possibilities for its control. Cold Spring Harb. Perspect. Med..

[132] Sajid M., Khan M.S.A., Cameotra S.S., Ahmad I. (2014). Drug Delivery Systems That Eradicate and/or Prevent Biofilm Formation. Antibiofilm Agents.

[133] Khan M.S.A., Alshehrei F., Al-Ghamdi S.B., Bamaga M.A., Al-Thubiani A.S., Alam M.Z. (2020). Virulence and biofilms as promising targets in developing antipathogenic drugs against candidiasis. Futur. Sci. OA.

[134] Qais F.A., Khan M.S., Ahmad I. (2018). Nanoparticles as quorum sensing inhibitor: Prospects and limitations. Biotechnol. Appl. Quor. Sens. Inhib..

[135] Srivastava P., Kowshik M. (2013). Mechanisms of metal resistance and homeostasis in Haloarchaea. Archaea.

[136] Salas-Orozco M., Niño-Martínez N., Martínez-Castañón G.A., Méndez F.T., Jasso M.E.C., Ruiz F. (2019). Mechanisms of resistance to silver nanoparticles in endodontic bacteria: A literature review. J. Nanomater..

[137] Zhang R., Carlsson F., Edman M., Hummelgård M., Jonsson B.G., Bylund D., Olin H. (2018). Escherichia coli Bacteria Develop Adaptive Resistance to Antibacterial ZnO Nanoparticles. Adv. Biosyst..

[138] Siemer S., Westmeier D., Barz M., Eckrich J., Wünsch D., Seckert C., Thyssen C., Schilling O., Hasenberg M., Pang C. (2019). Biomolecule-corona formation confers resistance of bacteria to nanoparticle-induced killing: Implications for the design of improved nanoantibiotics. Biomaterials.

[139] De Lima R., Seabra A.B., Durán N. (2012). Silver nanoparticles: A brief review of cytotoxicity and genotoxicity of chemically and biogenically synthesized nanoparticles. J. Appl. Toxicol. JAT.

[140] Hendry A.T., Stewart I.O. (1979). Silver-resistant Enterobacteriaceae from hospital patients. Can. J. Microbiol..

[141] Li X.Z., Nikaido H., Williams K.E. (1997). Silver-resistant mutants of Escherichia coli display active efflux of Ag^+^ and are deficient in porins. J. Bacteriol..

[142] Gupta A., Silver S. (1998). Silver as a biocide: Will resistance become a problem?. Nat. Biotechnol..

[143] Graves J.L., Tajkarimi M., Cunningham Q., Campbell A., Nonga H., Harrison S.H., Barrick J.E. (2015). Rapid evolution of silver nanoparticle resistance in Escherichia coli. Front. Genet..

[144] Hachicho N., Hoffmann P., Ahlert K., Heipieper H.J. (2014). Effect of silver nanoparticles and silver ions on growth and adaptive response mechanisms of Pseudomonas putida mt-2. FEMS Microbiol. Lett..

[145] Cui L., Wang X., Huang D., Zhao Y., Feng J., Lu Q., Pu Q., Wang Y., Cheng G., Wu M. (2020). CRISPR-cas3 of Salmonella upregulates bacterial biofilm formation and virulence to host cells by targeting quorum-sensing systems. Pathogens.

[146] Gholizadeh P., Köse Ş., Dao S., Ganbarov K., Tanomand A., Dal T., Aghazadeh M., Ghotaslou R., Rezaee M.A., Yousefi B. (2020). How CRISPR-Cas system could be used to combat antimicrobial resistance. Infect. Drug Resist..

[147] Valentin E., Bottomley A.L., Chilambi G.S., Harry E.J., Amal R., Sotiriou G.A., Rice S.A., Gunawan C. (2020). Heritable nanosilver resistance in priority pathogen: A unique genetic adaptation and comparison with ionic silver and antibiotics. Nanoscale.

[148] Faghihzadeh F., Anaya N.M., Astudillo-Castro C., Oyanedel-Craver V. (2018). Kinetic, metabolic and macromolecular response of bacteria to chronic nanoparticle exposure in continuous culture. Environ. Sci. Nano.

[149] Finley P.J., Norton R., Austin C., Mitchell A., Zank S., Durham P. (2015). Unprecedented silver resistance in clinically isolated Enterobacteriaceae: Major implications for burn and wound management. Antimicrob. Agents Chemother..

[150] Matuła K., Richter Ł., Janczuk-Richter M., Nogala W., Grzeszkowiak M., Peplińska B., Jurga S., Wyroba E., Suski S., Bilski H. (2019). Phenotypic plasticity of Escherichia coli upon exposure to physical stress induced by ZnO nanorods. Sci. Rep..

[151] Liu S.B., Ng A.K., Xu R., Wei J., Tan C.M., Yang Y.H., Chen Y.Y., Kang S., Pinault M., Pfefferle L.D. (2010). Antibacterial action of dispersed single-walled carbon nanotubes on Escherichia coli and Bacillus subtilis investigated by atomic force microscopy. Nanoscale.

[152] Baym M., Lieberman T.D., Kelsic E.D., Chait R., Gross R., Yelin I., Kishony R. (2016). Spatiotemporal microbial evolution on antibiotic landscapes. Science.

[153] Zhang Q., Lambert G., Liao D., Kim H., Robin K., Tung C.K., Pourmand N., Austin R.H. (2011). Acceleration of emergence of bacterial antibiotic resistance in connected microenvironments. Science.

[154] Moghadam M.T., Amirmozafari N., Shariati A., Hallajzadeh M., Mirkalantari S., Khoshbayan A., Jazi F.M. (2020). How phages overcome the challenges of drug resistant bacteria in clinical infections. Infect. Drug Resist..

[155] Brives C., Pourraz J. (2020). Phage therapy as a potential solution in the fight against AMR: Obstacles and possible futures. Palgrave Commun..

[156] Hay I.D., Lithgow T. (2019). Filamentous phages: Masters of a microbial sharing economy. EMBO Rep..

[157] Pirnay J.P. (2020). Phage Therapy in the Year 2035. Front. Microbiol..

[158] Wright A., Hawkins C.H., Änggård E.E., Harper D.R. (2009). A controlled clinical trial of a therapeutic bacteriophage preparation in chronic otitis due to antibiotic-resistant Pseudomonas aeruginosa; A preliminary report of efficacy. Clin. Otolaryngol..

[159] Leitner L., Ujmajuridze A., Chanishvili N., Goderdzishvili M., Chkonia I., Rigvava S., Chkhotua A., Changashvili G., McCallin S., Schneider M.P. (2020). Intravesical bacteriophages for treating urinary tract infections in patients undergoing transurethral resection of the prostate: A randomised, placebo-controlled, double-blind clinical trial. Lancet Infect. Dis..

[160] Speck P., Smithyman A. (2015). Safety and efficacy of phage therapy via the intravenous route. FEMS Microbiol. Lett..

[161] Aslam S., Lampley E., Wooten D., Karris M., Benson C., Strathdee S., Schooley R.T. (2020). Lessons learned from the first 10 consecutive cases of intravenous bacteriophage therapy to treat multidrug-resistant bacterial infections at a single center in the United States. Open Forum Infect. Dis..

[162] Voelker R. (2019). FDA Approves Bacteriophage Trial. JAMA.

[163] Cisek A.A., Dąbrowska I., Gregorczyk K.P., Wyżewski Z. (2017). Phage Therapy in Bacterial Infections Treatment: One Hundred Years after the Discovery of Bacteriophages. Curr. Microbiol..

[164] Gordillo Altamirano F.L., Barr J.J. (2019). Phage therapy in the postantibiotic era. Clin. Microbiol. Rev..

[165] Gazeev S. (2018). Fagterapi och dess Tillämpning inom Veterinärmedicin. Applications of Phage Therapy in Veterinary Medicine Applications of Phage Therapy in Veterinary Medicine.

[166] Smirnov D.D., Kapustin A.V., Yakimova E.A., Savinov V.A., Laishevtsev A.I. (2020). Perspectives of the use of bacteriophages in agriculture, food and processing industries. IOP Conf. Ser. Earth Environ. Sci..

[167] Endersen L., Coffey A. (2020). The use of bacteriophages for food safety. Curr. Opin. Food Sci..

[168] Summers W.C. (2012). The strange history of phage therapy. Bacteriophage.

[169] Lin D.M., Koskella B., Lin H.C. (2017). Phage therapy: An alternative to antibiotics in the age of multi-drug resistance. World J. Gastrointest. Pharmacol. Ther..

[170] Van Belleghem J.D., Dąbrowska K., Vaneechoutte M., Barr J.J., Bollyky P.L. (2019). Interactions between bacteriophage, bacteria, and the mammalian immune system. Viruses.

[171] Lehti T.A., Pajunen M.I., Skog M.S., Finne J. (2017). Internalization of a polysialic acid-binding Escherichia coli bacteriophage into eukaryotic neuroblastoma cells. Nat. Commun..

[172] Di Giovine M., Salone B., Martina Y., Amati V., Zambruno G., Cundari E., Failla C.M., Saggio I. (2001). Binding properties, cell delivery, and gene transfer of adenoviral penton base displaying bacteriophage. Virology.

[173] Weber-Dabrowska B., Zimecki M., Mulczyk M. (2000). Effective phage therapy is associated with normalization of cytokine production by blood cell cultures. Arch. Immunol. Ther. Exp..

[174] Park K., Cha K.E., Myung H. (2014). Observation of inflammatory responses in mice orally fed with bacteriophage T7. J. Appl. Microbiol..

[175] Szermer-Olearnik B., Boratyński J. (2015). Removal of endotoxins from bacteriophage preparations by extraction with organic solvents. PLoS ONE.

[176] Manohar P., Tamhankar A.J., Leptihn S., Ramesh N. (2019). Pharmacological and Immunological Aspects of Phage Therapy. Infect. Microbes Dis..

[177] Maciejewska B., Olszak T., Drulis-Kawa Z. (2018). Applications of bacteriophages versus phage enzymes to combat and cure bacterial infections: An ambitious and also a realistic application?. Appl. Microbiol. Biotechnol..

[178] Łusiak-Szelachowska M., Zaczek M., Weber-Dabrowska B., Miȩdzybrodzki R., Kłak M., Fortuna W., Letkiewicz S., Rogóz P., Szufnarowski K., Jończyk-Matysiak E. (2014). Phage neutralization by sera of patients receiving phage therapy. Viral Immunol..

[179] Górski A., Miedzybrodzki R., Borysowski J., Dabrowska K., Wierzbicki P., Ohams M., Korczak-Kowalska G., Olszowska-Zaremba N., Łusiak-Szelachowska M., Kłak M. (2012). Phage as a Modulator of Immune Responses. Practical Implications for Phage Therapy. Adv. Virus Res..

[180] Hess K.L., Jewell C.M. (2020). Phage display as a tool for vaccine and immunotherapy development. Bioeng. Transl. Med..

[181] Vukotic G., Obradovic M., Novovic K., Di Luca M., Jovcic B., Fira D., Neve H., Kojic M., McAuliffe O. (2020). Characterization, Antibiofilm, and Depolymerizing Activity of Two Phages Active on Carbapenem-Resistant Acinetobacter baumannii. Front. Med..

[182] Ferriol-González C., Domingo-Calap P. (2020). Phages for Biofilm Removal. Antibiotics.

[183] Tagliaferri T.L., Jansen M., Horz H.P. (2019). Fighting Pathogenic Bacteria on Two Fronts: Phages and Antibiotics as Combined Strategy. Front. Cell. Infect. Microbiol..

[184] Rodriguez-Gonzalez R.A., Leung C.Y., Chan B.K., Turner P.E., Weitz J.S. (2019). Quantitative Models of Phage-Antibiotics Combination Therapy. BioRxiv.

[185] Morrisette T., Kebriaei R., Morales S., Rybak M.J. (2020). Bacteriophage-Antibiotic Combinations: A Promising Alternative for Refractory Infections?. Contagion.

[186] Liu C.G., Green S.I., Min L., Clark J.R., Salazar K.C., Terwilliger A.L., Kaplan H.B., Trautner B.W., Ramig R.F., Maresso A.W. (2020). Phage-antibiotic synergy is driven by a unique combination of antibacterial mechanism of action and stoichiometry. MBio.

[187] Paczesny J., Richter Ł., Hołyst R. (2020). Recent progress in the detection of bacteria using bacteriophages: A review. Viruses.

[188] Banuelos S., Gulbudak H., Horn M.A., Huang Q., Nandi A., Ryu H., Segal R. (2020). Investigating the impact of combination phage and antibiotic therapy: A modeling study. BioRxiv.

[189] Górski A., Borysowski J., Międzybrodzki R. (2020). Phage therapy: Towards a successful clinical trial. Antibiotics.

[190] Peng H., Borg R.E., Dow L.P., Pruitt B.L., Chen I.A. (2020). Controlled phage therapy by photothermal ablation of specific bacterial species using gold nanorods targeted by chimeric phages. Proc. Natl. Acad. Sci. USA.

[191] Paczesny J., Bielec K. (2020). Application of bacteriophages in nanotechnology. Nanomaterials.

[192] Quispe-Tintaya W. (2017). Phage-Enabled Nanomedicine: From Probes to Therapeutics in Precision Medicine. Physiol. Behav..

[193] Labrie S.J., Samson J.E., Moineau S. (2010). Bacteriophage resistance mechanisms. Nat. Rev. Microbiol..

[194] Foster T.J. (2005). Immune evasion by staphylococci. Nat. Rev. Microbiol..

[195] Seed K.D. (2015). Battling Phages: How Bacteria Defend against Viral Attack. PLoS Pathog..

[196] Destoumieux-Garzón D., Duquesne S., Peduzzi J., Goulard C., Desmadril M., Letellier L., Rebuffat S., Boulanger P. (2005). The iron-siderophore transporter FhuA is the receptor for the antimicrobial peptide microcin J25: Role of the microcin Val11-Pro16 β-hairpin region in the recognition mechanism. Biochem. J..

[197] Vasu K., Nagaraja V. (2013). Diverse Functions of Restriction-Modification Systems in Addition to Cellular Defense. Microbiol. Mol. Biol. Rev..

[198] Goldfarb T., Sberro H., Weinstock E., Cohen O., Doron S., Charpak-Amikam Y., Afik S., Ofir G., Sorek R. (2015). BREX is a novel phage resistance system widespread in microbial genomes. EMBO J..

[199] Chaudhary K. (2018). BacteRiophage EXclusion (BREX): A novel anti-phage mechanism in the arsenal of bacterial defense system. J. Cell. Physiol..

[200] Ofir G., Melamed S., Sberro H., Mukamel Z., Silverman S., Yaakov G., Doron S., Sorek R. (2018). DISARM is a widespread bacterial defence system with broad anti-phage activities. Nat. Microbiol..

[201] Swarts D.C., Jore M.M., Westra E.R., Zhu Y., Janssen J.H., Snijders A.P., Wang Y., Patel D.J., Berenguer J., Brouns S.J.J. (2014). DNA-guided DNA interference by a prokaryotic Argonaute. Nature.

[202] Xiong L., Liu S., Chen S., Xiao Y., Zhu B., Gao Y., Zhang Y., Chen B., Luo J., Deng Z. (2019). A new type of DNA phosphorothioation-based antiviral system in archaea. Nat. Commun..

[203] Kronheim S., Daniel-Ivad M., Duan Z., Hwang S., Wong A.I., Mantel I., Nodwell J.R., Maxwell K.L. (2018). A chemical defence against phage infection. Nature.

[204] Phumyen A., Jantasorn S., Jumnainsong A., Leelayuwat C. (2014). Doxorubicin-conjugated bacteriophages carrying anti-MHC class I chain-related A for targeted cancer therapy in vitro. Onco. Targets. Ther..

[205] Lau R.K., Ye Q., Birkholz E.A., Berg K.R., Patel L., Mathews I.T., Watrous J.D., Ego K., Whiteley A.T., Lowey B. (2020). Structure and Mechanism of a Cyclic Trinucleotide-Activated Bacterial Endonuclease Mediating Bacteriophage Immunity. Mol. Cell.

[206] Millman A., Melamed S., Amitai G., Sorek R. (2020). Diversity and classification of cyclic-oligonucleotide-based anti-phage signalling systems. Nat. Microbiol..

[207] Hampton H.G., Watson B.N.J., Fineran P.C. (2020). The arms race between bacteria and their phage foes. Nature.

[208] Boyaval P., Moineau S., Romero D.A., Horvath P. (2007). Against Viruses in Prokaryotes. Science.

[209] Broniewski J.M., Chisnall M.A.W., Molin N., Westra E.R. (2021). The effect of Quorum sensing inhibitors on the evolution of CRISPR-based phage immunity in Pseudomonas aeruginosa. ISME J..

[210] Shehreen S., Chyou T.Y., Fineran P.C., Brown C.M. (2019). Genome-wide correlation analysis suggests different roles of CRISPR-Cas systems in the acquisition of antibiotic resistance genes in diverse species. Philos. Trans. R. Soc. B Biol. Sci..

[211] Trasanidou D., Gerós A.S., Mohanraju P., Nieuwenweg A.C., Nobrega F.L., Staals R.H.J. (2019). Keeping crispr in check: Diverse mechanisms of phage-encoded anti-crisprs. FEMS Microbiol. Lett..

[212] Landsberger M., Gandon S., Meaden S., Rollie C., Chevallereau A., Chabas H., Buckling A., Westra E.R., van Houte S. (2018). Anti-CRISPR Phages Cooperate to Overcome CRISPR-Cas Immunity. Cell.

[213] Gussow A.B., Park A.E., Borges A.L., Shmakov S.A., Makarova K.S., Wolf Y.I., Bondy-Denomy J., Koonin E.V. (2020). Machine-learning approach expands the repertoire of anti-CRISPR protein families. Nat. Commun..

[214] Matsumoto D., Tamamura H., Nomura W. (2020). A cell cycle-dependent CRISPR-Cas9 activation system based on an anti-CRISPR protein shows improved genome editing accuracy. Commun. Biol..

[215] Stanley S.Y., Borges A.L., Chen K.H., Swaney D.L., Krogan N.J., Bondy-Denomy J., Davidson A.R. (2019). Anti-CRISPR-Associated Proteins Are Crucial Repressors of Anti-CRISPR Transcription. Cell.

[216] Rostøl J.T., Marraffini L. (2019). (Ph)ighting Phages: How Bacteria Resist Their Parasites. Cell Host Microbe.

[217] Richter Ł., Księżarczyk K., Paszkowska K., Janczuk-Richter M., Niedziółka-Jönnson J., Gapiński J., Łoś M., Hołyst R., Paczesny J. (2021). Adsorption of bacteriophages on polypropylene labware affects reproducibility of phage research. Sci. Rep..

